# Examining the identification of age-related atrophy between T1 and T1 + T2-FLAIR cortical thickness measurements

**DOI:** 10.1038/s41598-019-47294-2

**Published:** 2019-08-02

**Authors:** Heidi Lindroth, Veena A. Nair, Casandra Stanfield, Cameron Casey, Rosaleena Mohanty, Daniel Wayer, Paul Rowley, Roger Brown, Vivek Prabhakaran, Robert D. Sanders

**Affiliations:** 1 Department of Anesthesiology, School of Medicine and Public Health, University of Wisconsin-Madison, Madison, WI USA; 2 School of Nursing, University of Wisconsin-Madison, Madison, WI USA; 3 Indiana University, School of Medicine. Division of Pulmonary, Sleep, and Critical Care Medicine. Center for Health Innovation and Implementation Science, Center for Aging Research, Regenstrief Institute, Indiana, USA

**Keywords:** Brain, Prognostic markers, Prognostic markers, Brain

## Abstract

Cortical thickness is traditionally derived from T1-weighted MRI images. Recent studies have shown an improvement in segmentation with the combination of T1 + T2-FLAIR images. MRI data from 54 adults (mean: 71 years, 65–81 years, 48% females) that are part of an ongoing cohort study were analyzed to investigate whether T1 + T2-FLAIR cortical thickness measurements were superior to those derived from T1-weighted images in identifying age-related atrophy. T1-weighted and T2-FLAIR MRI images were processed through FreeSurfer v6.0. Data was extracted using the Desikan-Killiany (DKT) atlas. FreeSurfer’s GUI QDEC examined age-related atrophy. Nonparametric tests, effect sizes, and Pearson correlations examined differences between T1-only and T1 + T2-FLAIR cortical thickness data. These analyses demonstrated that T1 + T2-FLAIR processed images significantly improved the segmentation of gray matter (chi-square *x*^2^, p < 0.05) and demonstrated significantly thicker cortical thickness means (p < 0.05) with medium to large effect sizes. Significant regions of age-related cortical atrophy were identified within the T1 + T2-FLAIR data (FDR corrected, p < 0.05). This is in contrast to the T1-only data where no regions survived FDR correction. In summary, T1 + T2-FLAIR data were associated with significant improvement in cortical segmentation and the identification of age-related cortical atrophy. Future studies should consider employing this imaging strategy to obtain cortical thickness measurements sensitive to age-related changes.

## Introduction

Cortical thickness is widely used to characterize grey matter changes with aging and disease. Cortical atrophy occurs as a natural process of aging as well as in different types of disease processes leading to focal or regional atrophy^1^. This atrophy equals a loss of neuron containing tissue and the connections between those neurons leading to functional and cognitive deficits^1,2^. Cortical thickness measurements may be used to identify relationships between patient-level clinical variables and regions of brain atrophy as well as predict those most likely to convert from mild cognitive impairment (MCI) to Alzheimer’s Disease (AD)^1,3–9^. Precise measurement of cortical thickness is critical to accurately identifying those that are likely to convert from MCI to AD, especially considering the monetary and societal burden of AD.

Cortical thickness measures are typically derived from T1-weighted anatomical magnetic resonance imaging (MRI) images. A popular high-resolution whole brain T1-weighted sequence is the three-dimension magnetization-prepared rapid gradient echo (MP-RAGE). It is a short sequence, with variable flip angle, and sensitive to magnetic field inhomogeneity that usually occurs at the interface between entities (e.g. tissue and air) that have different magnetic susceptibilities^10^. While this source has been documented to be reliable, segmentation errors are often identified when the dura layer is included in the gray matter measurements^11,12^. This is attributed to equal signal intensity from gray matter and dura, and varies across brain regions^11^. Traditionally, these segmentation errors are manually corrected, or images are discarded if there are significant errors. FreeSurfer^13–21^, a widely-used, open source, processing stream for surface-based cortical thickness measures, contains a multi-modal processing option using both T1- and T2-FLAIR (fluid-attenuated inversion-recovery) images intended to improve dural surface segmentation. In the FLAIR sequence, a conventional spin-echo sequence is prefaced by a 180 degree inversion pulse, and a relatively long inversion time, leading to a strongly T2-weighted image and suppressed CSF signal in the cortical or periventricular areas. This is especially useful for detecting lesions and for identifying hyperintense lesions that fall along the border of fluid containing spaces such as sulci or ventricles in the brain^10,22,23^. The added suppression of cerebrospinal fluid with FLAIR MR imaging and differences in signal intensity between gray matter and dura can be used to discriminate between brain regions with different morphologies of the cortical layers as well as assist with skull-stripping and removal of the dura matter from the image^11,24^. Precise derivation of cortical thickness measures depends on the correct segmentation of gray matter from the surrounding white matter and dura surfaces. Imprecise segmentation likely leads to inaccuracies in analysis and results, impacting the ability to accurately predict disease progression.

There are few studies to date that evaluate the difference between T1-only and T1 + T2-FLAIR processed images. These studies identified that multimodal imaging, using both T1-only and T1 + T2-FLAIR anatomical images decreased segmentation error and misidentification of tissue^11,12,24^. Further, voxel-based morphometry (VBM) analysis of T1 + T2-FLAIR data demonstrated higher correlation with age than T1-only data in a cohort of young to middle age participants^25^. Taken together, these studies suggest that T1 + T2-FLAIR derived cortical thickness measures lead to decreased segmentation errors and may increase the ability to identify age-related changes. To build on the VBM findings by Lindig *et al*.^25^, we hypothesized that surface-based T1 + T2-FLAIR cortical thickness measures would identify a larger area, and more regions, of age-related atrophy in a cohort of older adults. Further, we wanted to quantify the statistical differences between T1-only and T1 + T2-FLAIR surface-based data in terms of means, effect sizes, and correlations. Therefore, the purpose of the present study was to quantify the differences between T1-only and T1 + T2-FLAIR surface-based cortical thickness measures and to investigate the ability of T1 + T2-FLAIR data to identify age-related atrophy in a cohort of older adults, compared to T1-only data. These findings will inform future clinical research studies using cortical atrophy to assess current, or predict future, disease progression in older adults. Since multimodal imaging using both T1 + T2-FLAIR sequences is associated with increased cost and time spent in the scanner, it is important to investigate whether the potential gain in measurement precision is worth the additional time and money.

## Materials and Methods

**Figure 1:**
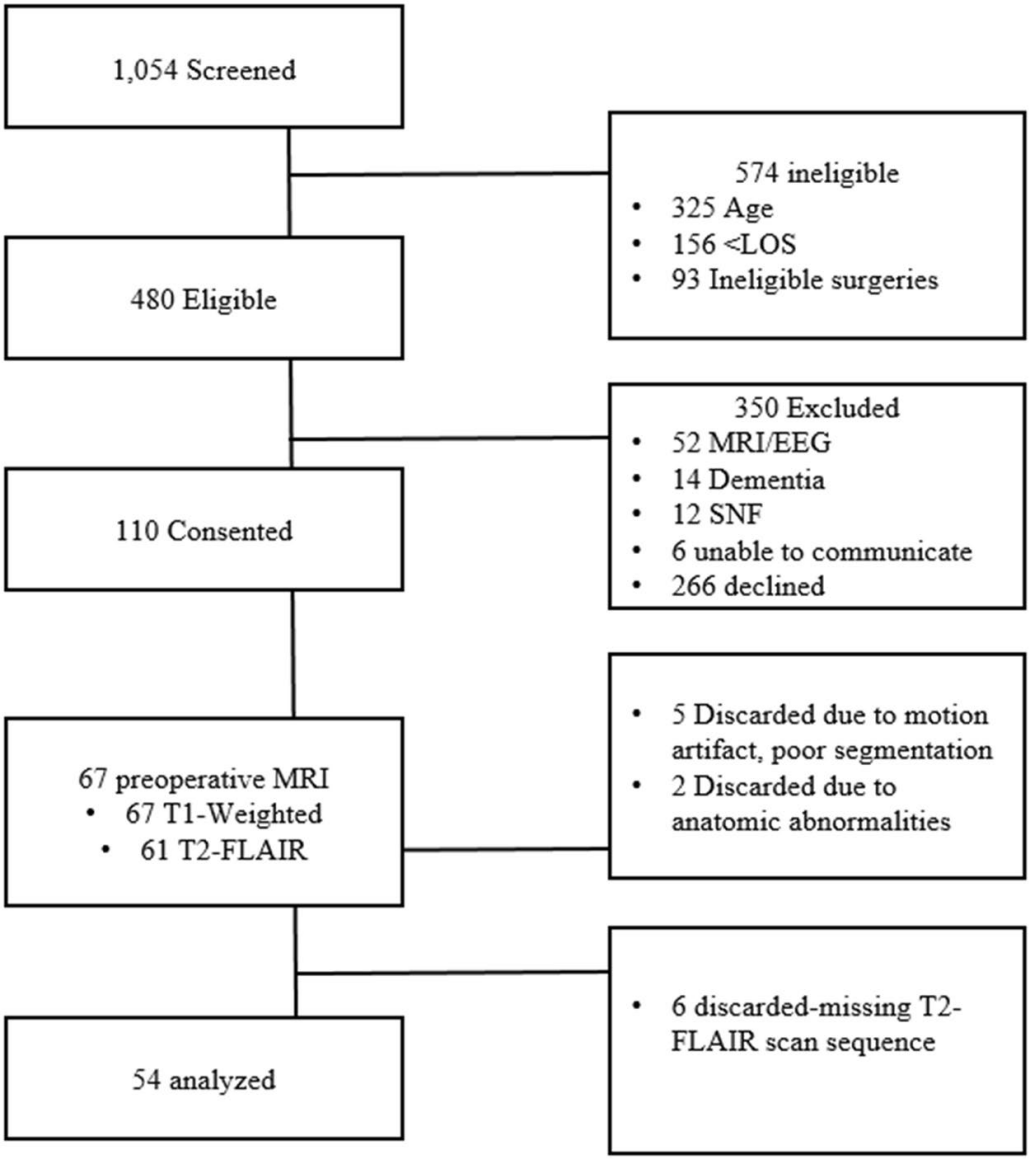
Study Flow.

This is a cross-sectional, descriptive analysis of sixty-seven participants who underwent a preoperative brain MRI as part of an ongoing perioperative cohort study registered with ClinicalTrials.gov (ref: NCT03124303, NCT01980511) and approved by the University of Wisconsin-Madison Institutional Review Board (#2015-0374). Seven MRI images were discarded due to motion artifact (5) or anatomical anomalies (2), and an additional six MRI images were discarded for an incomplete MRI sequences (either T1 or T2-FLAIR were not completed), for a final sample size of 54. The anatomical anomalies included abscess from prior brain infection (2). Figure 1 describes study flow.

All participants completed full written informed consent prior to study participation. Older adults, sixty-five years of age and older, scheduled to undergo a non-cardiac surgery with an estimated length of stay in the hospital of two days or greater following their surgery were eligible to participate in this study. Eligible participants were excluded if they had contraindications to an MRI, unable to communicate with research staff due to language or sensory barriers, had a documented history of dementia, and resided in a nursing home or assisted care facility. All methods were carried out in accordance with relevant regulations and guidelines.

### MRI acquisition and processing

MR scans were performed using 1.5 T and 3.0 T GE Discovery MR750 scanner (GE Healthcare; Waukesha, WI) using eight-channel phased array head coil. T1-weighted MPRAGE pulse sequence parameters were the following: Repetition time (TR): 2,530 ms, Echo time (TE): 3.09 ms, flip Angle: 10°, 256 × 256 matrix, 208 coronal slices, 1 mm isotropic resolution, total acquisition time = 4:07 minutes. High-resolution 3D FSE sagittal T2-weighted Cube FLAIR images were collected from each participant using the following sequence parameters: TR = 6000 ms; TE = 132 ms; Inversion Time (TI) = 1709 ms; Field of View = 25 × 25 × (118 × 0.16) cm resulting in a slice thickness of 0.16 cm; matrix size = 256 × 256 × 118; excitation flip angle α = 90°, with parallel imaging (Autocalibrating Reconstruction of Cartesian images, ARC), total acquisition time = 5:33 minutes. Surface-based, cortical thickness (mm) measures were obtained using a publicly available software package, FreeSurfer v6.0 (https://surfer.nmr.mgh.harvard.edu/). First T1-weighted MPRAGE images were processed using the well-documented recon-all processing stream, which includes motion correction, skull-stripping, registration, segmentation, smoothing, and parcellation mapping. Coronal slices were manually inspected and ranked on quality level by two independent raters. Regions such as the medial temporal lobe are best viewed in the coronal plane. Examination in this plane allows for accurate identification of any misclassification. The coronal plane was our starting point for all visual inspection, however, we also confirmed with the other two planes whenever needed, especially in cases where the initial quality ranking was 3 or higher (indicating poor quality). Quality rankings were assigned as follows. A score of zero indicated a high quality scan with 0–1 segmentation errors observed. A score of one indicated a good quality scan with 2 errors noted. Examples include the same continuous segmentation error over multiple slices (i.e. dura included in gray matter on slides 85–90 in right upper frontal lobe) or 2 different types of errors (1-dura including, 1-CSF inclusion). A score of two indicated a fair quality scan with 3–5 errors noted. A score of 3 indicated a poor quality scan with >6 errors. A score of 4 indicated severe damage and recommended removal of the processed image from the analysis. Supplementary Table S-1 lists the quality rankings given to each image. Seven images were discarded due to motion artifact, poor segmentation, or anatomical abnormalities. All images were then re-processed using T1 + T2-FLAIR multimodal recon-all processing stream. Cortical parcellation statistics were extracted using the Desikan-Killiany Atlas (DKT), which contains 68 regions, 34 per hemisphere^26^. Means, standard deviations, and data distribution were then evaluated. Following re-processing, segmentation was reviewed by two independent raters and images were re-ranked on their level of quality. Disagreement was resolved between the two independent raters by a neuroimaging expert (VN) and the mentoring author (RDS). Statistical analysis was performed to evaluate the differences in cortical thickness measurement between the T1-only recon-all images versus the T1 + T2-FLAIR recon-all images (n = 54). Cortical volumes and area measurements were not examined.

### Statistical analysis

Descriptive patient characteristics were evaluated using means ± standard deviations for continuous variables, frequency counts with percentages for categorical variables and data distributions were visually inspected. Cortical thickness measures were adjusted for scanner-type and intracranial volume (ICV). The ComBat harmonization correction tool was applied to account for inter-site scanner variation^27^. This tool performs well in small sample sizes and is able to maintain biologic variability while correcting for inter-site scanner variation. Quality rankings between T1-only and T1 + T2-FLAIR coronal slices were compared using the chi-square test. Pearson correlations were calculated between age and T1-only/T1 + T2-FLAIR DKT regions (mm). The difference between the correlation values (r) were calculated. The r values were transformed using the Fischer-Z transformation then the z-scores were tested using a two-tailed t-test to determine whether the change in the correlation values were significant^28^. FreeSurfer’s GUI interface, Query, Design, Estimate, Contrast (QDEC), was used to regress age onto T1-only and T1 + T2-FLAIR cortical thickness measurements, scanner was added as a covariate to adjust for scanner type^29^. The cortical surfaces were smoothed with a kernel of 10 mm using a surface-based smoothing procedure that averages data across neighboring cortical locations. In contrast to 3D volumetric smoothing, the use of surface based smoothing allows for the integration of information to regions limited by the area along the cortical mantle, and prevents combining of data across sulcal space and cerebrospinal fluid (Salat *et al*.^30^). False Discovery Rate (FDR) threshold of 0.05 was used to control for multiple comparisons. Chi-square test compared segmentation ratings. T1-only statistics and T1 + T2-FLAIR statistics were compared using Pearson correlations and Student’s t-test, both within- and between-subjects. If data was non-normally distributed, non-parametric Mann-Whitney U-test were performed. Effect sizes were calculated to demonstrate the magnitude of change, or difference, between the T1-only and T1 + T2-FLAIR cortical thickness measurements. Becker’s effect size calculation was used because of the dependent, or repeated, cortical thickness measurements^31,32^.

## Results

Fifty-four participants with a mean age of 71 (4.84) and 48% female were included in this cortical thickness analysis. Table 1 summarizes participant characteristics including a breakdown on the distribution of participants between scanners. Cortical thickness data from T1-only and T1 + T2-FLAIR processed images were extracted from FreeSurfer using the 68-region, 34-per hemisphere, DKT atlas then adjusted for scanner-type with the ComBat harmonization tool^26,27^.

**Table 1:** Participant characteristics.

Demographics	
Variable	Mean (SD) N = 54
Age (yrs)	71.74 ± 4.84
Education	1.74 ± 0.56
Sex	48% (female)
Scanner Type (**n**)	3T #1: 34
	3T #2: 14
	3T #3: 5
	1.5T #4: 1

This table displays the demographic characteristics of the sample size.

Education was defined as an ordinal variable: >12 years = 2, 12 years = 1, <12 years = 0.

Scanner types are defined by strength (3 Telsa (3T) vs 1.5 Telsa) (1.5T) and scanner number (1–4).

**Figure 2:**
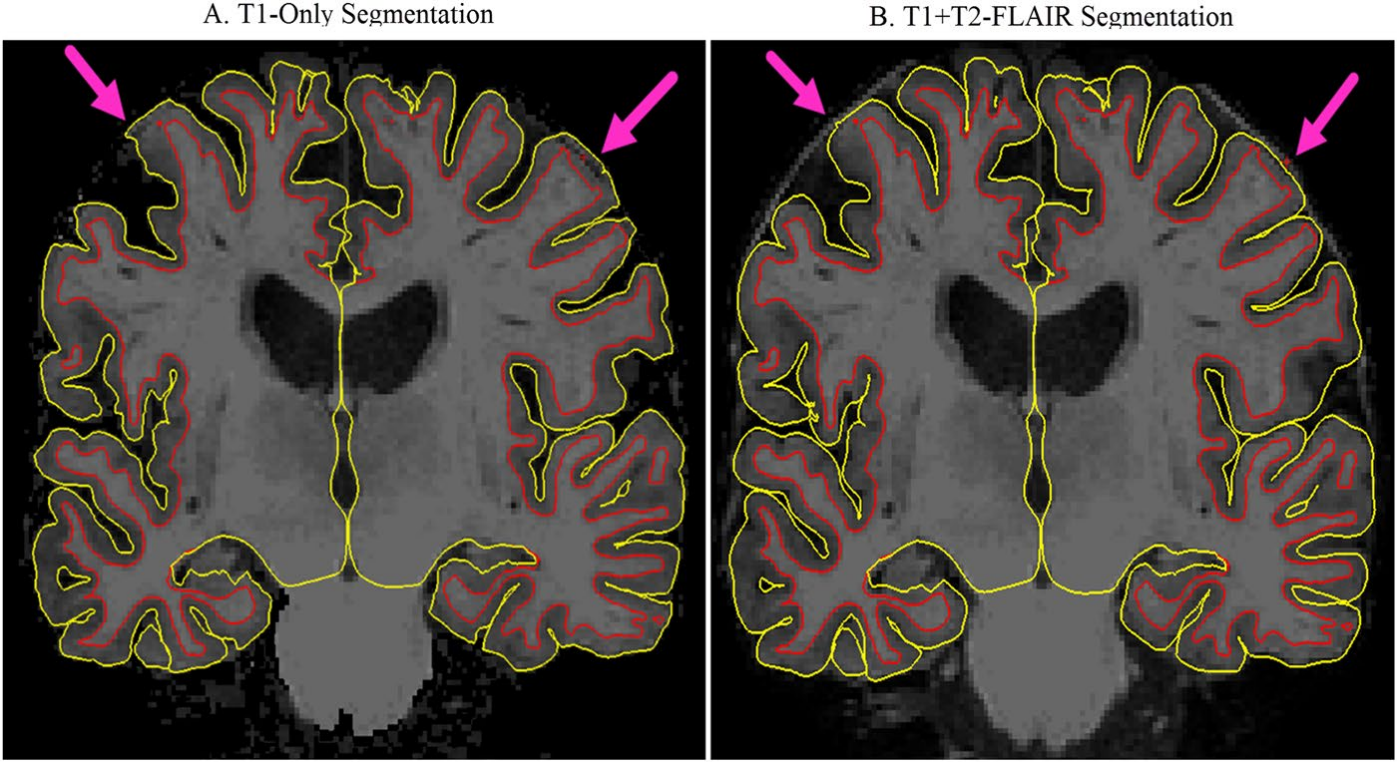
Illustrates the segmentation changes from the T1-only (**A**) to T1 + T2-FLAIR (**B**) processed MRI images. The red line represents the segmented white matter (light gray color) from the gray matter (darker gray color). The yellow line represents the gray matter segmentation from the dural and outlying brain material. In box A, the pink arrows point to segmentation overlap in the dura. In box B, the pink arrows point to the improved segmentation following the T1 + T2-FLAIR scan processing.

T1 + T2-FLAIR cortical thickness images demonstrated significant improvement in segmentation, (*x*^2^, p < 0.05), with 82% of the images ranking as “0 = high quality” or “1 = good quality”. This is contrasted to 55% of T1-only images categorized accordingly. Figure 2 demonstrates this improvement.

**Figure 3:**
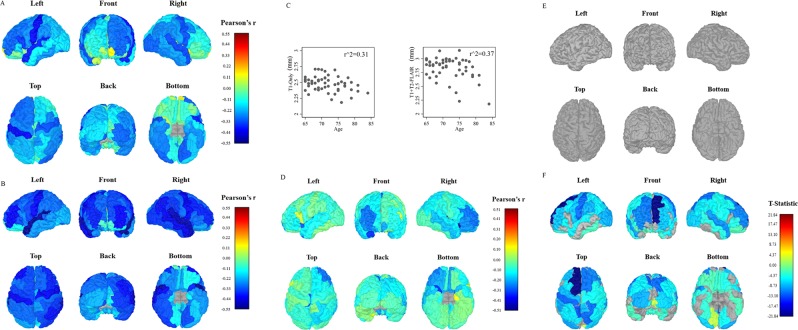
Illustrates the Pearson correlation (r) values between age and DKT regions (mm) (**A**–**D**) as well as T1-Only and T1 + T2-FLAIR DKT regions significantly associated with age (**E**,**F**). Brain regions in A represent the correlation values between T1-only DKT cortical thickness region means (mm) and age. Brain regions in (**B**) represent the correlation values between T1 + T2-FLAIR DKT cortical thickness region means (mm) and age. The scatterplots in (**C**) illustrate the Pearson correlation values between the left hemisphere DKT region superior frontal region (mm) and age in both T1-only (left) and T1 + T2-FLAIR (right) data. The r^2 value is in the upper right hand corner for each. Brain regions in D illustrate the difference between the age and DKT cortical thickness regions (n = 68) correlations ® in T1-only and T1 + T2-FLAIR data. Greater differences between the two Pearson r values are illustrated in deeper blue to red. Cortical thickness values were regressed on age as a focal predictor with scanner-type included as a covariate, using the general linear model framework. Resulting t-statistics per region were adjusted using a False Discovery Rate (FDR) significance correction procedure, with alpha set to 0.05, to account for the inflation of Type I error rate caused by multiple hypothesis testing. Brain regions E illustrate T1-only DKT analysis in which no regions were found to be significantly associated with age after FDR correction. Brain regions F displays T1 + T2-FLAIR DKT regions that survived FDR correction. Greater cortical atrophy regions are displayed with darker shades of blue. The left hemisphere Superior Frontal Region demonstrated the largest amount of atrophy and is pictured in the scatterplots (**C**).

Pearson correlations between age and T1-only DKT regions (mm) were largely negative (59/68 regions, r = −0.01 to −0.44) with 19 DKT regions showing a significant negative correlation with age. Pearson correlations between age and T1 + T2-FLAIR regions (mm) were all negative (68/68, r = −0.04 to −0.55) with 39 DKT regions showing a significant negative correlation with age. We then calculated the difference between T1-only and T1 + T2-FLAIR correlation values and test this difference for statistical significance. Three DKT regions had a statistically significant change in their age*DKT region (mm) correlation values; the left hemisphere paracentral lobule and posterior cingulate cortex and the right insula. These correlation values are illustrated in Fig. 3 and Supplementary Table S-2. Next, we examined bi-hemispheric age-related cortical atrophy using FreeSurfer’s GUI QDEC. In T1-only data, no vertices survived FDR correction at 0.05 unadjusted and scanner-adjusted. In T1 + T2-FLAIR data, FreeSurfer’s GUI QDEC identified 43 clusters representing 20034 surface vertices to survive FDR correction in the left hemisphere and 39 clusters representing 32216 surface vertices to survive FDR correction in the right hemisphere, (FDR corrected, p < 0.05). These results remained after adjustment for scanner-type and are shown in Fig. 3 and Supplementary Table S-3.

**Figure 4:**
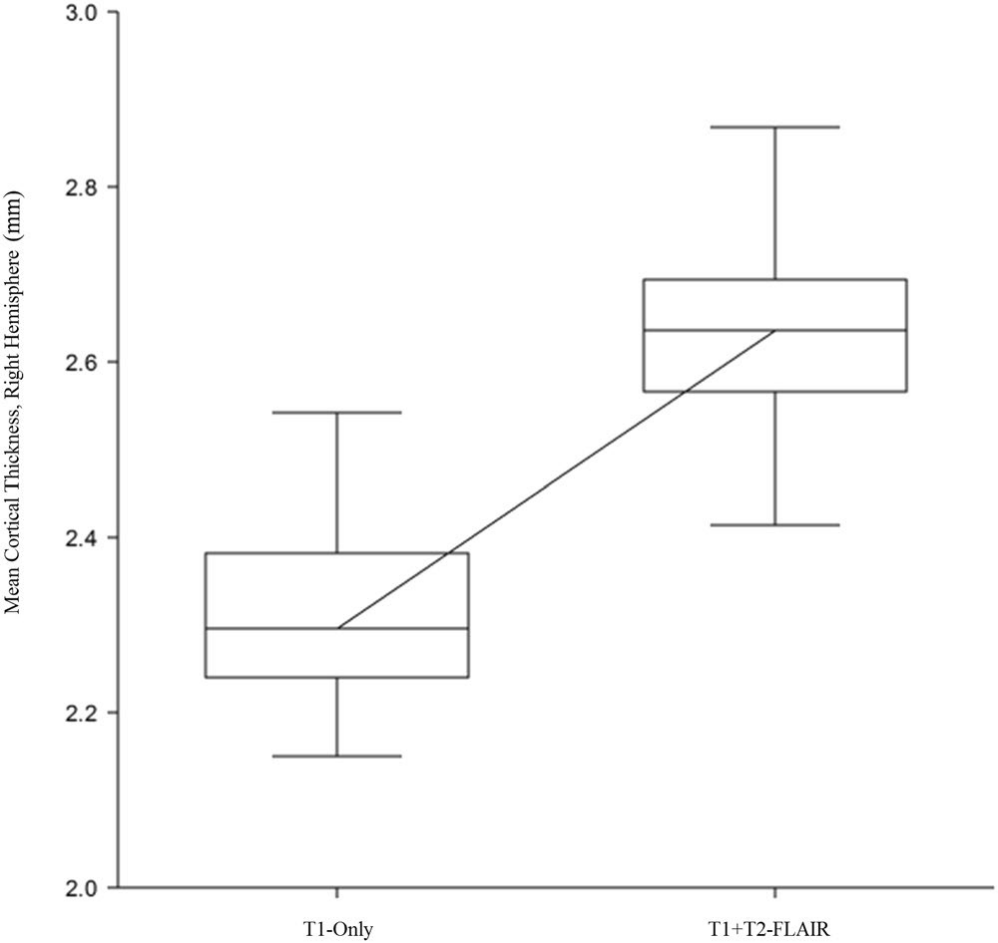
Illustrates the significant difference in overall cortical thickness means (mm) in the right hemisphere. The left hemisphere is not pictured and had similar significant differences observed. These are adjusted for scanner variance using the ComBat tool.

**Figure 5:**
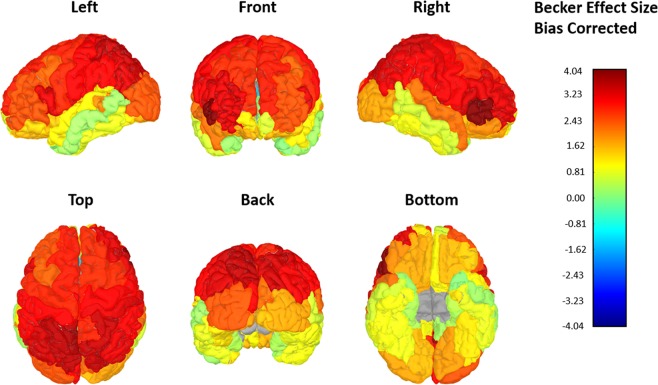
Illustrates the calculated Becker effect sizes. These were bias corrected and demonstrate the magnitude of change between the T1-only cortical thickness means and the T1 + T2-FLAIR cortical thickness mean per DKT region of interest. These data were adjusted for scanner variance using the ComBat tool. Increasing warm colors, yellow to red with red being the largest indicate increasing effect sizes. Green indicates minimal change, or a small effect size. Effect sizes are categorized as small, *d* = 0.2, medium, *d* = 0.5, large, *d* = 0.8.

T1 + T2-FLAIR cortical thickness means were significantly higher in 62 of 68 DKT regions (Mann Whitney U-test, p < 0.05) in both unadjusted and ComBat adjusted data, indicating thicker cortical measurements. One region, the bilateral caudal anterior cingulate cortices, demonstrated a significantly lower mean in T1 + T2-FLAIR data (p < 0.05). The four regions that did not demonstrate significantly different means were the middle temporal gyrus (T1: 2.65 mm ± 0.31 vs T1 + T2-FLAIR: 2.68 mm ± 0.19, p = 0.08) and the rostral anterior cingulate cortex (T1: 2.72 mm ± 0.21 vs T1 + T2-FLAIR: 2.74 ± 0.24, p = 0.45) in the left hemisphere and the temporal pole in bilateral hemispheres (LH-T1: 3.40 mm ± 0.28 vs T1 + T2-FLAIR: 3.42 mm ± 0.36, p = 0.42; RH-T1: 3.33 mm ± 0.28 vs T1 + T2-FLAIR: 3.39 mm ± 0.38, p = 0.08). Figure 4 illustrates the significant differences between T1-only and T1 + T2-FLAIR right hemisphere thickness means (the left hemisphere analysis showed the same result, is not pictured). Total ICV was not significantly different (p = 0.25). Medium-large Becker effect sizes (small, *d* = 0.2, medium, *d* = 0.5, large, *d* = 0.8) were observed in 61/68 DKT regions. These data are displayed in Table 2 and Fig. 5. Atrophy rate per year (mm/year) for both T1-only and T1 + T2-FLAIR DKT regions are displayed in Supplementary Table S-4.

**Table 2:** Describes overall mean thickness, effect sizes.

Means, Standard Deviations, and Becker Effect Size per hemisphere, per DKT Region								
DKT Region	LH				RH			
	T1 (mm) Mean ± SD	T1 + T2-FLAIR (mm) Mean ± SD	p-value	Effect Size	T1 (mm) Mean ± SD	T1 + T2-FLAIR (mm) Mean ± SD	p-value	Effect Size
Banks of superior temporal sulcus	2.32 ± 0.11	2.54 ± 0.17	<0.0001	1.97	2.42 ± 0.17	2.69 ± 0.21	<0.0001	1.57
Caudal Anterior Cingulate cortex	2.73 ± 0.31	2.34 ± 0.41	<0.0001	−1.24	2.60 ± 0.30	2.38 ± 0.35	0.0002	−0.72
Caudal Middle Frontal gyrus	2.33 ± 0.15	2.68 ± 0.22	<0.0001	2.3	2.32 ± 0.13	2.68 ± 0.19	<0.0001	2.73
Cuneus cortex	1.93 ± 0.12	2.30 ± 0.18	<0.0001	3.04	1.90 ± 0.13	2.20 ± 0.21	<0.0001	2.22
Entorhinal cortex	3.02 ± 0.33	3.17 ± 0.38	0.0004	0.45	3.16 ± 0.36	3.29 ± 0.39	0.0012	0.36
Fusiform gyrus	2.55 ± 0.15	2.74 ± 0.21	<0.0001	1.25	2.51 ± 0.18	2.67 ± 0.23	<0.0001	0.88
Inferior parietal lobule	2.28 ± 0.11	2.66 ± 0.17	<0.0001	3.41	2.33 ± 0.11	2.69 ± 0.19	<0.0001	3.23
Inferior temporal gyrus	2.49 ± 0.18	2.64 ± 0.27	<0.0001	0.82	2.47 ± 0.18	2.65 ± 0.22	<0.0001	0.93
Isthmus cingulate cortex	2.32 ± 0.19	2.49 ± 0.26	<0.0001	0.30	2.32 ± 0.19	2.51 ± 0.24	<0.0001	0.99
Lateral occipital cortex	2.05 ± 0.13	2.37 ± 0.20	<0.0001	2.43	2.09 ± 0.15	2.36 ± 0.23	<0.0001	1.77
Lateral orbitofrontal	2.34 ± 0.14	2.57 ± 0.20	<0.0001	1.62	2.29 ± 0.13	2.52 ± 0.20	<0.0001	1.74
Lingual gyrus	2.00 ± 0.14	2.26 ± 0.19	<0.0001	1.83	1.95 ± 0.13	2.16 ± 0.18	<0.0001	1.59
Middle temporal gyrus	2.65 ± 0.13	2.68 ± 0.19	0.0783	0.25	2.63 ± 0.15	2.73 ± 0.24	<0.0001	0.66
Orbitofrontal gyrus	2.34 ± 0.13	2.48 ± 0.24	<0.0001	1.06	2.37 ± 0.13	2.58 ± 0.20	<0.0001	1.59
Parahippocampal gyrus	2.61 ± 0.30	2.72 ± 0.36	0.0023	0.36	2.61 ± 0.25	2.70 ± 0.32	0.0061	0.35
Paracentral lobule	2.37 ± 0.12	2.76 ± 0.20	<0.0001	3.2	2.33 ± 0.13	2.71 ± 0.21	<0.0001	2.88
Pars opercularis	2.38 ± 0.10	2.72 ± 0.20	<0.0001	3.35	2.32 ± 0.12	2.72 ± 0.21	<0.0001	3.37
Pars orbitalis	2.41 ± 0.19	2.69 ± 0.28	<0.0001	1.45	2.38 ± 0.16	2.70 ± 0.26	<0.0001	1.97
Pars triangularis	2.28 ± 0.14	2.63 ± 0.21	<0.0001	2.46	2.23 ± 0.10	2.64 ± 0.19	<0.0001	4.04
Pericalcarine cortex	1.70 ± 0.12	2.08 ± 0.18	<0.0001	3.12	1.63 ± 0.14	1.97 ± 0.18	<0.0001	2.39
Postcentral gyrus	2.04 ± 0.11	2.38 ± 0.23	<0.0001	3.05	2.02 ± 0.10	2.35 ± 0.22	<0.0001	3.25
Posterior cingulate cortex	2.43 ± 0.18	2.49 ± 0.24	0.0310	0.33	2.37 ± 0.17	2.43 ± 0.23	0.0331	0.35
Precentral gyrus	2.39 ± 0.13	2.73 ± 0.22	<0.0001	2.79	2.37 ± 0.11	2.71 ± 0.21	<0.0001	3.05
Precuneus	2.27 ± 0.11	2.68 ± 0.16	<0.0001	3.67	2.30 ± 0.13	2.67 ± 0.17	<0.0001	2.81
Rostral Anterior Cingulate Cortex	2.72 ± 0.21	2.74 ± 0.24	0.4461	0.09	2.79 ± 0.23	2.87 ± 0.28	0.0353	0.34
Rostral middle frontal gyrus	2.19 ± 0.12	2.49 ± 0.16	<0.0001	2.46	2.15 ± 0.11	2.52 ± 0.15	<0.0001	3.32
Superior frontal	2.48 ± 0.12	2.83 ± 0.20	<0.0001	2.88	2.46 ± 0.13	2.82 ± 0.19	<0.0001	2.73
Superior parietal lobule	2.12 ± 0.11	2.51 ± 0.21	<0.0001	2.58	2.13 ± 0.11	2.51 ± 0.20	<0.0001	3.50
Superior temporal gyrus	2.53 ± 0.13	2.70 ± 0.19	<0.0001	1.14	2.52 ± 0.13	2.81 ± 0.24	<0.0001	2.20
Supramarginal	2.33 ± 0.12	2.70 ± 0.21	<0.0001	3.04	2.33 ± 0.12	2.40 ± 0.20	<0.0001	3.04
Frontal pole	2.52 ± 0.27	2.70 ± 0.36	0.0003	0.66	2.44 ± 0.23	2.72 ± 0.33	<0.0001	1.24
Temporal pole	3.40 ± 0.28	3.42 ± 0.36	0.4158	0.07	3.33 ± 0.28	3.39 ± 0.38	0.0783	0.21
Transverse temporal gyrus	2.27 ± 0.18	2.61 ± 0.28	<0.0001	1.86	2.30 ± 0.20	2.66 ± 0.32	<0.0001	1.77
Insula	2.75 ± 0.13	2.96 ± 0.19	<0.0001	1.59	2.76 ± 0.14	2.99 ± 0.20	<0.0001	1.62

This table details the calculated means, standard deviations, the p-value between T1-only and T1 + T2-FLAIR cortical thickness measures (millimeters) per DKT region, and the Becker effect size (small: *d* = 0.2; medium: *d* = 0.5; large: *d* = 0.8). The Becker effect size is used when dependent samples are compared.

Within-subject T1-only versus T1 + T2-FLAIR cortical thickness means were examined. The 34 DKT regions per hemisphere per participant were averaged for a left and right hemisphere mean. Fifty-three participants demonstrated bilaterally significantly thicker T1 + T2-FLAIR cortical thickness means (Mann Whitney U-test, p < 0.05).

**Figure 6:**
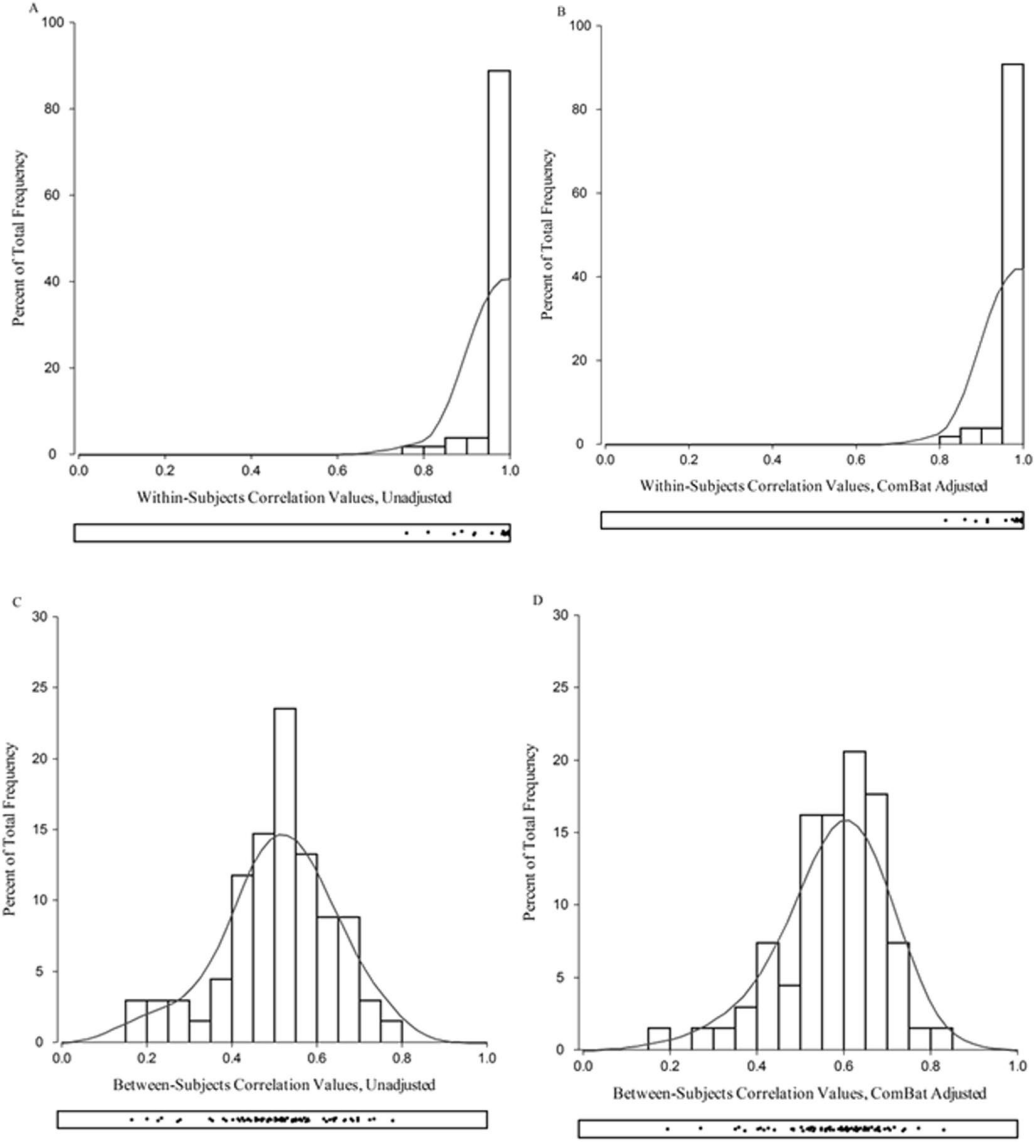
Illustrates the r^2^ correlation values within- and between-subjects. Plots A and B show the within-subject correlations, the right and left hemisphere means were compared between T1-only and T1 + T2-FLAIR data per participant. Plot A is unadjusted and Plot B is ComBat adjusted data. Plots C and D display the between-subject correlations per DKT region (68 total), right and left hemisphere DKT regions means were compared. Plot C is unadjusted and Plot D is ComBat adjusted data. Density plots are below each histogram to further illustrate the distribution.

Sixty-four of 68 unadjusted T1-only DKT regions significantly correlated with T1 + T2-FLAIR DKT regions (r^2^, p < 0.05). The four exceptions were the right hemisphere pars triangularis (r^2^ = 0.20, p = 0.15), postcentral gyrus (r^2^ = 0.23, p = 0.09), rostral anterior cingulate cortex (r^2^ = 0.17, p = 0.23), and the rostral middle frontal cortex (r^2^ = 0.23, p = 0.10). These pairwise correlations improved with ComBat adjustment, 67/68 T1-only DKT regions significantly correlated with T1 + T2-FLAIR DKT regions (r^2^, p < 0.05). The right hemisphere rostral anterior cingulate cortex was the one exception (r^2^ = 0.19, p = 0.16). Within-subject correlations were performed to examine agreement between T1-only to T1 + T2-FLAIR DKT cortical thickness data at the participant level. Averaging the 34 DKT regions per hemisphere per participant produced T1-only and T1 + T2-FLAIR means, these were then correlated. All 54 participants demonstrated r^2^ values >0.75 between their T1-only and T1 + T2-FLAIR derived cortical thickness measures, (p < 0.05). Histograms displaying both within- and between-subject correlations are illustrated in Fig. 6.

Two sensitivity analyses were completed. To evaluate the effectiveness of ComBat Harmonization tool, scanner type was regressed out of each DKT region mean using a general linear model. These means were compared to ComBat adjusted cortical thickness means using standard t-tests. Four DKT regions were identified to be significantly different (p < 0.05) between the scanner-type regression adjustment and the ComBat harmonization data. The second sensitivity analysis evaluated pairwise DKT T1-only and T1 + T2-FLAIR correlations by removing a consistent outlier. When between-subject correlations were repeated, the resulting r^2^ values were largely unchanged. The largest observed change was in the left hemisphere, Banks of Superior Temporal Sulcus (r^2^:0.27–0.18, p = 0.92).

## Discussion

This analysis demonstrated significant differences between T1-only and T1 + T2-FLAIR surface-based cortical thickness measures both in terms of segmentation accuracy and sensitivity to age-related atrophy in a narrow age-range of older adults. T1 + T2-FLAIR, but not T1-only, cortical thickness measures identified age-related atrophy in this cohort of elderly patients with a narrow age range (65–81 years old). Although significantly correlated, T1 + T2-FLAIR processed images had overall significantly thicker DKT cortical thickness means. These between-subject correlations improved with scanner adjustment using the ComBat method. Most regions demonstrated large effect sizes indicating a sizeable magnitude of change between T1-only and T1 + T2-FLAIR cortical thickness measurements. These differences and improvement in cortical thickness measurement combine to demonstrate that multimodal imaging, using T1 + T2-FLAIR, provides a more complete measurement of surface-based cortical thickness in older adults.

The combination of T1 + T2-FLAIR anatomical images yielded significant improvement in the segmentation of gray matter from the dura layer, resulting in significantly higher cortical thickness means per DKT region. Our results are congruent with previous research that investigated the ratio of signal intensity in gray to white matter with aging tissue. The signal intensity ratio was reported to be higher on FLAIR images than on T1-weighted images, with the cause of this change attributed to factors such as age-related decrease in water content, and an increase in the iron concentration etc.^30^ Choi *et al*.^33^ observed that the ratio of GM and WM thicknesses was the highest on the FLAIR, and that gray matter signal intensity decreased with aging, leading to reduced contrast between gray and white matter boundries^33^. Salat *et al*.^30^ also reported that signal intensity between gray and white matter decreased with age leading to reduced contrast between tissue classes. This is a regional phenomenon, mostly affecting the superior frontal, precentral, postcentral, occipital, medial frontal, and superior temporal regions^11,30^. The ability of T2-FLAIR to enhance signal intensity from gray matter, and decrease signal intensity from CSF, leading to improved contrast, likely contributed to the overall, higher cortical thickness means particularly in the four regions (pars triangularis, rostral middle gyrus, postcentral, rostral anterior cingulate) that did not significantly correlate with T1-only data. Adjusting for scanner effect did improve these correlations, however, their overall means are unchanged and demonstrate large effect sizes between T1-only and T1 + T2-FLAIR data. Two of these regions, the rostral middle gyrus and the postcentral gyrus, were identified to be significantly associated with age-related atrophy in the T1 + T2-FLAIR cortical thickness measures. Therefore, T1 + T2-FLAIR may be better at detecting gray matter in these regions in older adults.

Our findings that the addition of T2-FLAIR improved segmentation accuracy are in congruence with previous studies that examined the difference between T1-only and T1 + T2-FLAIR segmentation and tissue classification, using different processing programs^11,12,24,25^. Viviani *et al*.^11^ and Lindig *et al*.^25^ identified that multimodal imaging increased accuracy through the differentiation of the dura layer and vessels from gray matter to improve segmentation, as well as an improved signal intensity^11,25^. This improvement likely results from the different pulse sequences used by T1-only and T2-FLAIR anatomical scans to identify different brain tissue and cerebrospinal fluid. T1-only images typically have a shorter pulse sequence in contrast to T2-FLAIR images, which have a longer pulse sequence and together with suppression of the signal from the CSF and higher T2 weighting, lead to an increased sensitivity to pathology and a differentiation from the dura layer^11,24^. The FLAIR has a specific advantage in that it is an inversion-recovery pulse sequence specifically designed to reduce the signal intensity from CSF and brain parenchyma, thus enhancing the gray matter-white matter contrast^10,22,23^.

Our finding that surface-based cortical thickness means increased using T1 + T2-FLAIR images is in contrast to previous studies using voxel-based morphometry (VBM)^11,25^. There are important differences between our current analysis and previous analyses that make direct comparisons difficult. First, the current study examined surface-based cortical thickness measures using FreeSurfer in contrast to Statistical Parametric Mapping based VBM approach The methods used to derive surface-based cortical thickness measures and volume based measures differ in measurement and the definition of reference points used to calculate the measurement^19^. These differences lead to varying cortical thickness measurement values within the same brain region and in turn, limit comparisons between the two methods^34^. Additionally, the current analysis examined a cohort of older adults with a mean age of seventy-two versus the previous studies focused on a younger cohort, with the mean age below 50 rendering a strict comparison between the methods challenging. This study identified that multimodal imaging, using T1 + T2-FLAIR, reduced segmentation errors and increased the amount of gray matter detected in surface-based cortical thickness measures.

Scanner adjustment using the ComBat method did improve the correlations between T1-only and T1 + T2-FLAIR data. Further, the data distributions and standard deviations were centered closer to the mean, indicating that the ComBat method did reduce the noise in the data that was introduced by using different scanners. This is important for future studies that use more than one scanner to consider, as the ComBat method is simple to apply and likely results in an enhanced distribution of the data leading to accurate measurement.

### Strengths and limitations

The present study contributes to current literature by examining statistical differences between T1-only and T1 + T2-FLAIR cortical thickness derived measures and evaluates the ability to identify age-related atrophy in a narrow range of older adults. The present study is limited by sample size. However, this also demonstrates the importance of using a sensitive tool to detect age-related atrophy. Secondly, we did not attempt to adjust for other atrophy related covariates hat may accumulate with age, rather we are attempting to identify any atrophy. It is important to note that our patients were healthy enough to be planning major surgery and did not include patients with dementia. An additional limitation to this analysis is the cross-sectional design, precluding the ability to examine atrophy change over time.

### Recommendations for future research

#### Use of T1 versus T1 + T2-FLAIR

Multimodal imaging, T1 + T2-FLAIR, improved surface-based cortical thickness measurements by reducing segmentation error and increasing the signal intensity of the gray matter. Future studies should consider the addition of T2-FLAIR images as it appears more sensitive to changes in cortical thickness associated with age. This addition would increase scan time as well as processing time; however, depending on the population and area of study, the potential advantages reported in this analysis may surpass the impediment of time as reduced segmentation error and increased signal intensity may lead to increased precision in the measurement of surface-based cortical thickness measurements.

## Conclusion

This cross-sectional, descriptive neuroimaging study identified that multimodal imaging, T1 + T2-FLAIR, improved the accuracy of surface-based cortical thickness measures by reducing segmentation errors and increasing signal intensity. Future studies should consider the use of multimodal imaging to improve surface-based cortical thickness measurement.

## Supplementary information

Supplementary Material

## Data Availability

Datasets generated during and/or analyzed during the current study are available from the corresponding author on reasonable request.
