# Supplementary material for: Examining the identification of age-related atrophy between T1 and T1 + T2-FLAIR cortical thickness measurements
Source: Sci Rep. 2019 Aug 2;9:11288. doi: 10.1038/s41598-019-47294-2 (PMC6677836; doi:10.1038/s41598-019-47294-2)
Supplement: Supplementary file 1 — Supplementary Material [file 41598_2019_47294_MOESM1_ESM.docx]

**Examining the identification of age-related atrophy between T1 and T1+T2-FLAIR cortical thickness measurements**

**Supplementary Information**

Heidi Lindroth PhD, RN;^1,2,3^ Veena A. Nair PhD;^1^ Casandra Stanfield AA;^1,2^ Cameron Casey BA, BS;^1^ Rosaleena Mohanty MS;^1^ Daniel Wayer BS;^1^ Paul Rowley BS;^1^ Roger Brown PhD;^2^ Vivek Prabhakaran MD;^1^ Robert D. Sanders PhD MD;^1*^

^1^Department of Anesthesiology, School of Medicine and Public Health, University of Wisconsin-Madison, Madison WI; ^2^School of Nursing, University of Wisconsin-Madison, Madison, WI; ^3^Department of Medicine, School of Medicine-Center for Aging Research, Indiana University, Indianapolis, IN @minipixie26;

**Work performed at:** University of Wisconsin-Madison, School of Medicine and Public Health

***Corresponding author**: Robert D. Sanders, Department of Anesthesiology, University of Wisconsin-Madison, School of Medicine and Public Health, 600 Highland Avenue, B6/319 CSC Madison WI 53792-3272, Telephone: 608-262-6469 Fax 608-263-0575 Madison, USA. Email: robert.sanders@wisc.edu

| Table S-1 details the quality rankings performed on image slices by two independent raters. | | | |
| --- | --- | --- | --- |
| T1-Only Images | | T1+T2-FLAIR Images | |
| Grade | Description  Per Slice # | Grade | Description  Per Slice # |
| 2 | Red line crosses over dura layer (175-178) (152-166) (132-150) | 1 | Minimal fix to dural overlap. |
| 4 | Mislabeled upper L of screen? (186-189), (55-86 major fix upper R). Poor skull stripping. | 4 | Reprocessed. Still a terrible scan. - discard. |
| 0 | No errors | 0 | No errors. |
| 2 | (60-62) minor, (62-68) spots in the cerebellum minor | 1 | Reprocessed. Edges (60-62) are improved but have some poor differentiation. |
| 2 | (156-162) bring red line in R side, (152-153) bring red line in L side | 0 | No errors. |
| 3 | (120-162) bring red line in R side, (153-161) Bring red line in L side, (60) mislabeled spot at the top | 0 | No errors. |
| 4 | (160-164) bring red line in R side, (157-158) bring red line in L side, (122-146) is the bottom portion mislabeled? | 2 | Image improved. Discarded due to temporal lobe. |
| 2 | (164-165) Red line R side, (130-132) mislabeled L upper | 1 | Image improved. (164-165) includes less dura than T1-only, minimal overlap. CSF segmentation errors <2. |
| 3 | (151-162) miss labeled upper R, (54-55) red line L upper, (46-47) upper R mislabeled. | 0 | Reprocessed- no errors. |
| 0 | No errors | 0 | No errors |
| 3 | Overall poor image quality. Slide 63 on R side - hole in WM. Overall grainy, poor quality image. Slice 145 on right. | 0 | No errors |
| 1 | Slide 76 includes CSF.  Slide 176, Right side, red line in WM | 0 | No errors |
| 3 | Overall dura segmentation error onto dura surface on R and L hemispheres. Bilateral frontal lobes. 161-109=minor infringements on dura surface. 109 has white matter in dura surface-minor. | 2 | Less than 5 dura infringements, now is graded as 2 instead of 3. |
| 4 | Blurry – movement artifact? | 1 | Discard. Poor quality. |
| 0 | No errors | 0 | No errors |
| 1 | Slice 49-43 Left inner lower section has overlapping red line into cerebellum. | 1 | Slide 43-49 fixed. New error on slice 91 Left lower, misplaced red line into hippocampal area, CSF captured. |
| 0 | No errors | 0 | No errors |
| 2 | Dural layer has minimal overlap throughout. | 1 | Small defect, lower left inner lobe slice 161-CSF included. All other dural overlap corrected. |
| 1 | 147-144 Right overlap into dura layer, upper lobe | 0 | No error |
| 2 | Left dura layer on upper frontal lobe is incorrect (144, 137-140, 96) | 0 | No error |
| 1 | 111 R upper lobe white/gray matter into dura layer | 0 | No error |
| 3 | 151 segmentation is messy-multiple infringements then images become grainy as slice #s decrease. Slice 140 has an anomaly of white/gray matter segmentation in upper R lobe. Image improves at 105 through 50 | 2 | Image is improved, less segmentation error into dura. Now graded a 2. |
| 1 | 151-149 L upper lobe, white/gray matter overlap into dura section. | 1 | Small dural overlap on Slice 151. |
| 1 | 120-113 R upper lobe overlap into dura | 0 | No error |
| 2 | 169 L upper lobe white/gray matter into dura. 137-103 dura overlap, both hemispheres | 1 | Minimal dural overlap 150-170 |
| 0 | No error | 0 | No error |
| 0 | No error | 0 | No error |
| 3 | 153-137 Poor white matter segmentation, grainy image. 97 bilateral inner lower lobes, gray matter segmentation in middle of white. Slice 46-43 R lower gray matter is segmented into cerebellum. CSF included in gray matter segmentation. | 1 | Segmentation greatly improved |
| 3 | 126 lower temporal lobes have segmentation infringements. 119 Bilateral upper infringement on dura layer. 107-105 L upper infringement on dura layer, white and gray matter. 51-107 bilateral upper lobe dura layer interference. | 1 | Minimal dural overlap slice 50, 62, 100-160. |
| 3 | 161 bilateral upper lobe infringement on dura layer - Overall image quality is good but the dura layer is overlapping with gray matter in several places. | 1 | Minimal dura overlap throughout, slice 161 is improved. |
| 1 | Slice 142 has small white matter overlap into dura layer. | 1 | Slice 142-white matter overlap is decreased but still present. CSF included in segmentation in 2 areas on left hemisphere |
| 0 | No errors | 0 | No errors |
| 1 | 105-115 small dura overlap on R upper | 0 | No errors |
| 2 | 141-131 bilateral upper hemispheres overlap with dura layer in places. 78-71 missing R upper point of gray matter. | 0 | No errors |
| 1 | Minimal dura overlap throughout. | 0 | No errors |
| 1 | 89,102,108 dura overlap bilateral front | 1 | 1=almost perfect. Slice 139 lower left inner lobe has anomaly with segmentation of white/gray matter, CSF included. |
| 0 | No errors | 0 | No errors |
| 1 | Bilateral lower hippocampal area, white/gray matter overlap-minimal. CSF included in segmentation. | 1 | Slice 137, 144 lower inner lobes have some segmentation overlap |
| 2 | 136-163 upper left lobe is misclassified - likely where abscess was removed |  | Discarded due to anatomical anomaly |
| 1 | Overall quality of scan seems lower than most, grainy images at points. No dura overlap. | 0 | Grainy images improved with addition of FLAIR |
| 0 | No errors | 0 | No errors. Discarded due to anatomical anomaly in front lobe |
| 1 | 125, 139 dura overlap | 0 | No errors |
| 2 | 79-94, 135-142. Minimal dura overlap, bilateral | 0 | No errors |
| 1 | 173 R lower lobe overlap | 0 | No errors |
| 1 | 110 slide-ringing in upper lobe? Movement? | 2 | Slice 78-69 left lower lobe has segmentation overlap into cerebellum. Overall image quality is not better than T1-only image |
| 3 | Dura overlap throughout, primarily on upper hemisphere, bilateral. | 2 | Moderate improvement on dura overlap |
| 3 | 122 R upper dura overlap. 139-50 dura overlap in place | 1 | Slice 122 is fixed. 50-139 have decreased dura overlap. |
| 1 | 156 - ringing or lines? Movement? | 1 | Slice 156 is improved with minimal segmentation error |
| 2 | 154 lower left lobe-move red line in. 142 L upper lobe-misclassification? 131 bilateral lower lobes-segmentation. 113-100 upper left lobe - missing piece? 81-77 Right frontal lobe-upper-missing? CSF included. | 0 | No errors |
| 1 | 106-87 R upper lobe lateral dura overlap | 0 | No errors |
| 1 | Slice 106 has anomalies bilateral lower inner lobes. CSF included. | 1 | Slice 106 improved. CSF not included |
| 4 | Discard. Poor quality scan. Possible motion during scan. | 4 |  |
| 3 | Dura layer overlap throughout | 2 | Dura layer overlap decreased throughout scan. |
| 4 | Discard. Poor quality scan. Possible motion during scan. | 4 |  |
| 1 | Slice 39-43 lower right dura overlap | 0 | No errors |
| 3 | Slice 147 has bilateral, lower dura overlap. Mild dura overlap throughout | 2 | The mild dura overlap throughout was corrected with FLAIR scan |
| 3 | Slice 129 significant dural overlap bilateral, 133 right gray/white matter overlap, slice 139 bottom middle (red?), slice 159 on R temporal horn, 120s have a lot of dural overlap, 63, 57. CSF included. | 1 | All slices improved with dura overlap. |
| 2 | Slices 130-105 have minor dural overlap in upper hemispheres. | 1 | Slice 52 has dural overlap on left, minimal. |
| 2 | Slice 146, upper left. Slice 140, upper right, 130-133, upper right. Slice 84, top. | 1 | Minimal dura overlap. Dura overlap observed in Slices 84-146 improved. |
| 1 | Slice 117 on left hemisphere, upper has dura overlap | 0 | No errors |
| 4 | Discard. Scanner motion. | 4 |  |
| 0 | No errors | 0 | No errors |
| Total | 0=10, 1=20, 2=13, 3=13, 4=5 | Total | 0=30, 1=19, 2=7, 3=0, 4=5 |
| Table S-1 describes the quality rankings performed on each image slice. This is an overall quality ranking score, taking all slices and errors into consideration.  The scoring system is as follows:   - 0 = 0-1 segmentation error, - 1 = 2 errors. These errors could be the same continuous error over multiple slices (i.e. dura included in grey matter on slices 85-90 in right upper frontal lobe) or 2 different types of errors (1-dura inclusion, 1-CSF inclusion), - 2= 3-5 segmentation errors, - 3= moderate segmentation error with dura overlap throughout (>6 errors), - 4= poor quality, discard. | | | |

| Table S-2 – Pearson correlations per hemisphere, between age and each DKT Region (68 total) | | | | | | | | | |  |
| --- | --- | --- | --- | --- | --- | --- | --- | --- | --- | --- |
|  | **LH** | | | | **RH** | | | | |  |
| DKT Region | | **T1**  **(r)** | **T1+T2-FLAIR (r)** | **Difference in r values** | **T1**  **(r)** | **T1+T2-FLAIR**  **(r)** | | **Difference in r values** | |  |
| Banks of superior temporal sulcus | | -0.22 | -0.32* | -0.10 | -0.12 | -0.39* | | -0.27 | |  |
| Caudal Anterior Cingulate cortex | | -0.01 | -0.08 | -0.07 | 0.15 | -0.07 | | -0.22 | |  |
| Caudal Middle Frontal gyrus | | -0.31* | -0.31* | 0.00 | -0.28* | -0.39* | | -0.11 | |  |
| Cuneus cortex | | -0.13 | -0.27 | -0.14 | -0.06 | -0.18 | | -0.12 | |  |
| Entorhinal cortex | | -0.26 | -0.14 | 0.12 | -0.06 | -0.18 | | -0.12 | |  |
| Fusiform gyrus | | -0.21 | -0.20 | 0.01 | -0.29* | -0.34* | | -0.05 | |  |
| Inferior parietal lobule | | -0.22 | -0.31* | -0.09 | -0.28* | -0.41* | | -0.13 | |  |
| Inferior temporal gyrus | | -0.27 | -0.26 | 0.01 | -0.28* | -0.35* | | -0.07 | |  |
| Isthmus cingulate cortex | | -0.05 | -0.20 | -0.15 | -0.10 | -0.12 | | -0.02 | |  |
| Lateral occipital cortex | | -0.19 | -0.24 | -0.05 | -0.32* | -0.37* | | -0.05 | |  |
| Lateral orbitofrontal | | -0.09 | -0.12 | -0.03 | -0.06 | -0.28* | | -0.22 | |  |
| Lingual gyrus | | -0.33* | -0.35* | -0.02 | -0.11 | -0.13 | | -0.02 | |  |
| Middle temporal gyrus | | -0.16 | -0.27 | -0.11 | -0.25 | -0.45* | | -0.20 | |  |
| Orbitofrontal gyrus | | -0.02 | -0.25 | -0.23 | -0.11 | -0.19 | | -0.08 | |  |
| Parahippocampal gyrus | | -0.29* | -0.29* | 0.00 | -0.20 | -0.24 | | -0.04 | |  |
| Paracentral lobule | | -0.04 | -0.35* | -0.31* | -0.05 | -0.21 | | -0.16 | |  |
| Pars opercularis | | -0.37* | -0.29* | -0.08 | -0.16 | -0.31* | | -0.15 | |  |
| Pars orbitalis | | -0.01 | -0.13 | -0.12 | -0.01 | -0.25 | | -0.24 | |  |
| Pars triangularis | | -0.20 | -0.35* | -0.15 | -0.06 | -0.31* | | -0.25 | |  |
| Pericalcarine cortex | | -0.05 | -0.20 | -0.15 | 0.05 | -0.04 | | -0.09 | |  |
| Postcentral gyrus | | -0.44* | -0.44* | 0.00 | -0.25 | -0.31* | | -0.06 | |  |
| Posterior cingulate cortex | | 0.12 | -0.34* | -0.46* | 0.03 | -0.17 | | -0.20 | |  |
| Precentral gyrus | | -0.28* | -0.36* | -0.08 | -0.34* | -0.44* | | -0.10 | |  |
| Precuneus | | -0.21 | -0.31* | -0.10 | -0.27* | -0.26 | | 0.01 | |  |
| Rostral Anterior Cingulate Cortex | | 0.03 | -0.08 | -0.11 | -0.06 | | -0.09 | | -0.03 | |
| Rostral middle frontal gyrus | | -0.31* | -0.42* | -0.10 | -0.11 | | -0.36* | | -0.28 | |
| Superior frontal | | -0.31* | -0.37* | -0.06 | -0.18 | | -0.32* | | -0.14 | |
| Superior parietal lobule | | -0.23 | -0.31* | -0.08 | -0.17 | | -0.34* | | -0.17 | |
| Superior temporal gyrus | | -0.40* | -0.55* | -0.15 | -0.35* | | -0.51* | | -0.16 | |
| Supramarginal | | -0.28* | -0.31* | -0.03 | -0.27* | | -0.37* | | -0.10 | |
| Frontal pole | | 0.16 | -0.07 | -0.23 | 0.05 | | -0.06 | | -0.11 | |
| Temporal pole | | -0.19 | -0.39* | -0.20 | -0.07 | | -0.28* | | -0.35 | |
| Transverse temporal gyrus | | -0.17 | -0.36* | -0.19 | -0.25 | | -0.30* | | -0.05 | |
| Insula | | 0.02 | -0.30* | -0.32 | 0.15 | | -0.36 | | -0.51* | |
| This table details the Pearson correlations between age and DKT region (mm) in both T1-only then T1+T2-FLAIR data. The difference between the two correlation coefficients (r) were calculated and are displayed. Significance <0.05 is illustrated with an asterisk * next to the numerical value. | | | | | | | | | |  |

| Table S-3: Results from FreeSurfers GUI QDEC. T1+T2-FLAIR cortical thickness regions associated with age and controlled for scanner-type. Regions surviving FDR 0.05 correction are listed. | | | | | | |
| --- | --- | --- | --- | --- | --- | --- |
| Cluster No. | **LH** | | | **RH** | | |
|  | **Area Name** | **Max**  **t-value** | **Size (mm^2^)** | **Area name** | **Max**  **t-value** | **Size (mm^2^)** |
| 1 | Superior temporal gyrus | -6.3642 | 1507.00 | Superior temporal gyrus | -6.2022 | 1481.33 |
| 2 | Postcentral gyrus | -5.8955 | 1813.88 | Superior frontal | -5.1920 | 1256.79 |
| 3 | Precuneus | -4.3868 | 272.24 | Caudal Middle Frontal gyrus | -4.7527 | 3227.37 |
| 4 | Orbitofrontal gyrus | -4.3850 | 579.30 | Insula | -4.3927 | 1617.04 |
| 5 | Supramarginal gyrus | -4.3452 | 415.90 | Inferior parietal lobule | -4.3180 | 1447.27 |
| 6 | Superior frontal | -4.2277 | 426.08 | Pars triangularis | -4.2270 | 614.46 |
| 7 | Lateral occipital cortex | -3.9295 | 230.00 | Superior temporal gyrus | -4.2196 | 563.86 |
| 8 | Caudal middle frontal gyrus | -3.9261 | 662.01 | Middle temporal gyrus | -4.1188 | 455.93 |
| 9 | Superior frontal | -3.9164 | 303.03 | Paracentral lobule | -3.9531 | 179.97 |
| 10 | Postcentral gyrus | -3.8082 | 322.57 | Medial Orbitofrontal | -3.6214 | 83.29 |
| 11 | Precuneus | -3.7753 | 141.63 | Fusiform gyrus | -3.5760 | 858.64 |
| 12 | Precentral gyrus | -3.5151 | 286.65 | Rostral middle frontal gyrus | -3.5314 | 616.32 |
| 13 | Superior parietal lobule | -3.4806 | 211.75 | Lateral occipital cortex | -3.4980 | 1051.75 |
| 14 | Inferior parietal lobule | -3.4675 | 402.90 | Precuneus | -3.4483 | 89.62 |
| 15 | Temporal pole | -3.3815 | 148.86 | Superior parietal lobule | -3.3851 | 215.04 |
| 16 | Rostral middle frontal gyrus | -3.3018 | 280.32 | Superior frontal | -3.1986 | 245.72 |
| 17 | Superior frontal | -3.1489 | 234.69 | Superior frontal | -3.1565 | 105.07 |
| 18 | Superior parietal lobule | -3.0813 | 123.11 | Superior parietal lobule | -3.1093 | 133.97 |
| 19 | Superior frontal | -3.0295 | 157.52 | Superior parietal lobule | -3.0857 | 203.32 |
| 20 | Lateral occipital cortex | -3.0014 | 288.51 | Supramarginal gyrus | -3.0449 | 395.77 |
| 21 | Inferior parietal lobule | -2.9945 | 65.94 | Inferior parietal lobule | -3.0327 | 99.05 |
| 22 | Medial Orbitofrontal | -2.9705 | 202.02 | Precentral gyrus | -2.8765 | 451.86 |
| 23 | Banks of Superior  Temporal Sulcus | -2.9453 | 35.01 | Rostral middle frontal gyrus | -2.8426 | 93.05 |
| 24 | Posterior cingulate cortex | -2.7681 | 14.38 | Entorhinal cortex | -2.7353 | 117.18 |
| 25 | Precentral gyrus | -2.7500 | 77.92 | Precuneus | -2.7238 | 76.34 |
| 26 | Postcentral gyrus | -2.7448 | 55.32 | Postcentral gyrus | -2.5352 | 102.92 |
| 27 | Pars triangularis | -2.7296 | 72.42 | Superior parietal lobule | -2.5264 | 53.95 |
| 28 | Supramarginal gyrus | -2.7186 | 71.90 | Lingual gyrus | 2.4904 | 65.45 |
| 29 | Rostral middle frontal gyrus | -2.6999 | 33.45 | Supramarginal gyrus | -2.3919 | 167.27 |
| 30 | Pars triangularis | -2.6516 | 125.72 | Rostral middle frontal gyrus | -2.3842 | 82.55 |
| 31 | Lingual gyrus | -2.6337 | 66.49 | Lateral  Orbitofrontal gyrus | -2.3179 | 17.70 |
| 32 | Superior frontal | -2.5691 | 42.09 | Precentral gyrus | -2.2225 | 38.92 |
| 33 | Precuneus | -2.5548 | 29.76 | Rostral middle frontal gyrus | -2.1181 | 11.59 |
| 34 | Superior frontal | -2.5428 | 77.65 | Insula | -2.1165 | 10.18 |
| 35 | Inferior temporal gyrus | -2.5123 | 86.38 | Lateral occipital cortex | -2.1144 | 17.62 |
| 36 | Caudal middle frontal gyrus | -2.4528 | 51.55 | Precuneus | -2.1075 | 9.20 |
| 37 | Lateral occipital cortex | -2.4143 | 47.29 | Pars orbitalis | -2.0796 | 10.31 |
| 38 | Superior frontal | -2.4012 | 55.05 | Lateral occipital cortex | -2.0508 | 5.89 |
| 39 | Precuneus | -2.3913 | 7.43 | Postcentral gyrus | -2.0156 | 0.36 |
| 40 | Supramarginal gyrus | -2.3885 | 9.30 |  |  |  |
| 41 | Lingual gyrus | -2.3584 | 57.45 |  |  |  |
| 42 | Banks of Superior  Temporal Sulcus | -2.3462 | 37.62 |  |  |  |
| 43 | Precentral gyrus | -2.3305 | 19.75 |  |  |  |
| 44 | Rostral middle frontal gyrus | -2.2578 | 7.06 |  |  |  |
| Table S-3 displays the results of FreeSurfer’s GUI QDEC analysis. Age and scanner-type were entered as covariates. The DKT regions listed survived False Discovery Rate (FDR) correction at the 0.05 level. This analysis was completed with T1-only DKT cortical thickness measures, no regions survived FDR correction. | | | | | | |

| Cortical Thickness Means | 65-74 yo | 75-84 yo | Significance |
| --- | --- | --- | --- |
| T1-Only, L Hemisphere | 2.35 | 2.28 | 0.02 |
| T1-Only, R Hemisphere | 2.33 | 2.28 | 0.06 |
|  |  |  |  |
| T1+T2-FLAIR L Hemisphere | 2.64 | 2.53 | 0.02 |
| T1+T2-FLAIR R Hemisphere | 2.63 | 2.53 | 0.05 |


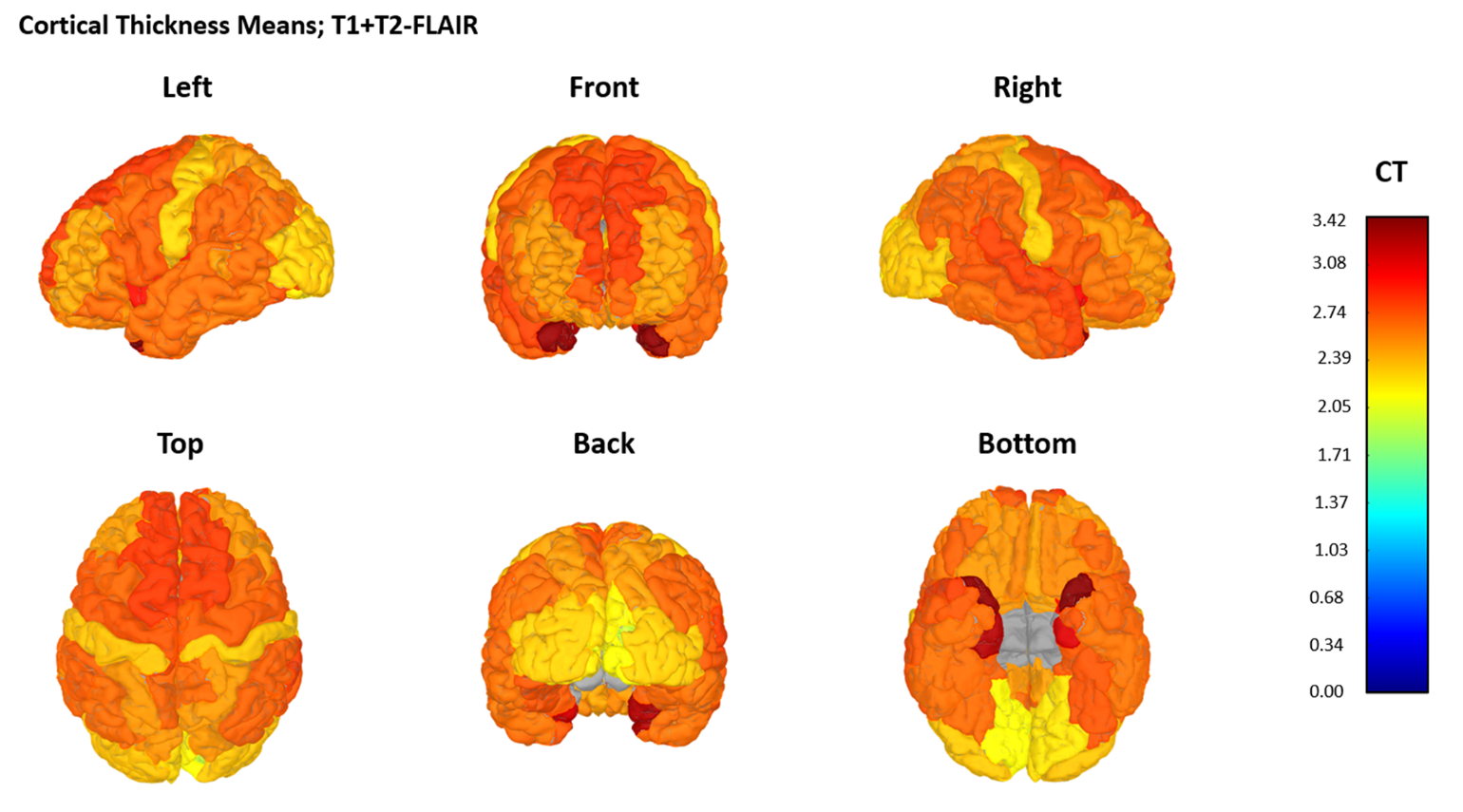

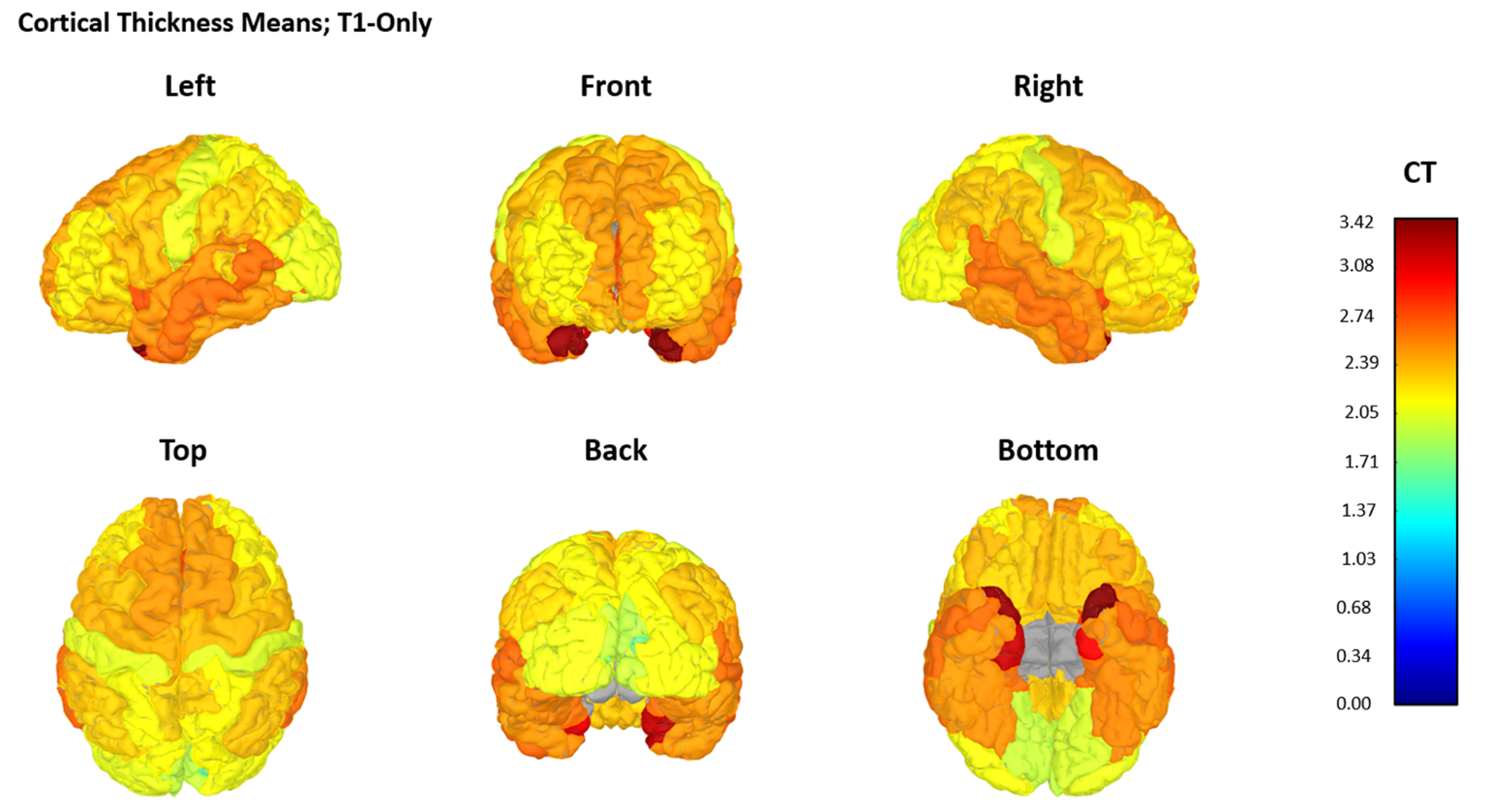


| **Table S-4.** Mean Atrophy Rates per DKT Region | | | | |
| --- | --- | --- | --- | --- |
|  | T1-Only | | T1 + T2-FLAIR | |
| DKT Region | Atrophy Rate  (mean, mm/year) | Std. Err. | Atrophy Rate  (mean, mm/year) | Std. Err. |
| Left Banks of superior temporal sulcus | -0.005 | 0.003 | -0.011 | 0.005 |
| Left Caudal anterior cingulate cortex | -0.003 | 0.008 | -0.007 | 0.011 |
| Left Caudal middle frontal gyrus | -0.010 | 0.004 | -0.014 | 0.006 |
| Left Cuneus cortex | -0.003 | 0.003 | -0.010 | 0.005 |
| Left Entorhinal cortex | -0.011 | 0.009 | -0.011 | 0.010 |
| Left Fusiform gyrus | -0.007 | 0.004 | -0.008 | 0.006 |
| Left Inferior parietal lobule | -0.005 | 0.003 | -0.011 | 0.005 |
| Left Inferior temporal gyrus | -0.009 | 0.005 | -0.013 | 0.007 |
| Left Isthmus cingulate cortex | -0.003 | 0.005 | -0.011 | 0.007 |
| Left Lateral occipital cortex | -0.004 | 0.003 | -0.010 | 0.006 |
| Left Lateral orbitofrontal | -0.002 | 0.004 | -0.005 | 0.006 |
| Left Lingual gyrus | -0.010 | 0.004 | -0.013 | 0.005 |
| Left Medial orbitofrontal gyrus | 0.001 | 0.004 | -0.013 | 0.007 |
| Left Middle temporal gyrus | -0.004 | 0.003 | -0.010 | 0.005 |
| Left Parahippocampal gyrus | -0.016 | 0.008 | -0.021 | 0.010 |
| Left Paracentral lobule | -0.003 | 0.003 | -0.014 | 0.005 |
| Left Pars opercularis | -0.007 | 0.003 | -0.011 | 0.005 |
| Left Pars orbitalis | 0.000 | 0.005 | -0.007 | 0.008 |
| Left Pars triangularis | -0.006 | 0.004 | -0.015 | 0.005 |
| Left Pericalcarine cortex | 0.000 | 0.003 | -0.007 | 0.005 |
| Left Postcentral gyrus | -0.010 | 0.003 | -0.020 | 0.006 |
| Left Posterior cingulate cortex | 0.003 | 0.005 | -0.016 | 0.006 |
| Left Precentral gyrus | -0.008 | 0.003 | -0.016 | 0.006 |
| Left Precuneus | -0.004 | 0.003 | -0.010 | 0.004 |
| Left Rostral anterior cingulate cortex | -0.001 | 0.006 | -0.004 | 0.007 |
| Left Rostral middle frontal gyrus | -0.007 | 0.003 | -0.014 | 0.004 |
| Left Superior frontal | -0.007 | 0.003 | -0.014 | 0.005 |
| Left Superior parietal lobule | -0.006 | 0.003 | -0.013 | 0.006 |
| Left Superior temporal gyrus | -0.010 | 0.003 | -0.023 | 0.005 |
| Left Supramarginal | -0.006 | 0.003 | -0.012 | 0.005 |
| Left Frontal pole | 0.007 | 0.007 | -0.005 | 0.010 |
| Left Temporal pole | -0.010 | 0.007 | -0.029 | 0.009 |
| Left Transverse temporal gyrus | -0.009 | 0.005 | -0.022 | 0.008 |
| Left Insula | 0.001 | 0.004 | -0.012 | 0.005 |
| Right Banks of superior temporal sulcus | -0.004 | 0.005 | -0.017 | 0.005 |
| Right Caudal anterior cingulate cortex | 0.008 | 0.008 | -0.005 | 0.010 |
| Right Caudal middle frontal gyrus | -0.007 | 0.003 | -0.015 | 0.005 |
| Right Cuneus Cortex | -0.002 | 0.004 | -0.008 | 0.006 |
| Right Entorhinal Cortex | 0.002 | 0.010 | -0.014 | 0.010 |
| Right Fusiform gyrus | -0.009 | 0.005 | -0.016 | 0.006 |
| Right Inferior parietal lobule | -0.006 | 0.003 | -0.016 | 0.005 |
| Right Inferior temporal gyrus | -0.009 | 0.004 | -0.016 | 0.006 |
| Right Isthmus cingulate cortex | -0.005 | 0.005 | -0.006 | 0.007 |
| Right Lateral occipital cortex | -0.008 | 0.004 | -0.017 | 0.006 |
| Right Lateral orbitofrontal gyrus | -0.001 | 0.004 | -0.012 | 0.005 |
| Right Lingual gyrus | -0.004 | 0.004 | -0.005 | 0.005 |
| Right Medial orbitofrontal gyrus | -0.002 | 0.003 | -0.008 | 0.006 |
| Right Middle temporal gyrus | -0.006 | 0.004 | -0.022 | 0.006 |
| Right Parahippocampal gyrus | -0.008 | 0.007 | -0.016 | 0.009 |
| Right Paracentral lobule | -0.002 | 0.003 | -0.009 | 0.006 |
| Right Pars opercularis | -0.002 | 0.003 | -0.014 | 0.006 |
| Right Pars orbitalis | -0.001 | 0.004 | -0.013 | 0.007 |
| Right Pars triangularis | 0.000 | 0.003 | -0.012 | 0.005 |
| Right Pericalcarine cortex | 0.002 | 0.004 | 0.001 | 0.005 |
| Right Postcentral gyrus | -0.005 | 0.003 | -0.014 | 0.006 |
| Right Posterior cingulate cortex | -0.001 | 0.005 | -0.008 | 0.006 |
| Right Precentral gyrus | -0.007 | 0.003 | -0.019 | 0.005 |
| Right Precuneus | -0.007 | 0.003 | -0.009 | 0.004 |
| Right Rostral anterior cingulate cortex | 0.007 | 0.007 | -0.005 | 0.008 |
| Right Rostral middle frontal gyrus | -0.002 | 0.003 | -0.012 | 0.004 |
| Right Superior frontal | -0.005 | 0.003 | -0.012 | 0.005 |
| Right Superior parietal lobule | -0.005 | 0.003 | -0.014 | 0.005 |
| Right Superior temporal gyrus | -0.009 | 0.003 | -0.025 | 0.006 |
| Right Supramarginal | -0.007 | 0.003 | -0.015 | 0.005 |
| Right Frontal pole | 0.002 | 0.006 | -0.004 | 0.009 |
| Right Temporal pole | -0.004 | 0.007 | -0.022 | 0.010 |
| Right Transverse temporal gyrus | -0.011 | 0.005 | -0.020 | 0.008 |
| Right Insula | 0.004 | 0.004 | -0.014 | 0.005 |
| Table S-4 displays the mean atrophy rate per year (mm/year) in each DKT region (34 per hemisphere). | | | | |
